# Metastatic Carcinomas at the Episiotomy Site: A Systematic Literature Review

**DOI:** 10.3390/cancers17172801

**Published:** 2025-08-27

**Authors:** Andrea Palicelli, Gabriele Tonni, Federica Torricelli, Beatrice Melli, Vincenza Ylenia Cusenza, Sandra Martinelli, Eleonora Zanetti, Alessandra Bisagni, Magda Zanelli, Maria Paola Bonasoni, Teresa Rossi, Lucia Mangone, Venus Damaris Medina-Illueca, Maurizio Zizzo, Andrea Morini, Giuseppe Broggi, Rosario Caltabiano, Serena Salzano, Francesca Sanguedolce, Nektarios I. Koufopoulos, Ioannis Boutas, Aleksandra Asaturova, Chiara Casartelli, Sara Rubagotti, Matteo Crotti, Lorenzo Aguzzoli, Vincenzo Dario Mandato

**Affiliations:** 1 Pathology Unit, Azienda USL-IRCCS di Reggio Emilia, 42123 Reggio Emilia, Italy; eleonora.zanetti@ausl.re.it (E.Z.); alessandra.bisagni@ausl.re.it (A.B.); magda.zanelli@ausl.re.it (M.Z.); mariapaola.bonasoni@ausl.re.it (M.P.B.); 2 Clinical and Experimental Medicine PhD Program, University of Modena and Reggio Emilia, 41121 Modena, Italy; chiara.casartelli@ausl.re.it; 3 Department of Obstetrics and Neonatology, Azienda USL-IRCCS di Reggio Emilia, 42123 Reggio Emilia, Italy; gabriele.tonni@ausl.re.it; 4 Laboratory of Translational Research, Azienda USL-IRCCS di Reggio Emilia, 42123 Reggio Emilia, Italy; federica.torricelli@ausl.re.it (F.T.); teresa.rossi@ausl.re.it (T.R.); 5 Molecular Pathology Unit, Azienda USL-IRCCS di Reggio Emilia, 42123 Reggio Emilia, Italy; beatrice.melli@ausl.re.it (B.M.); vincenzaylenia.cusenza@ausl.re.it (V.Y.C.); sandra.martinelli@ausl.re.it (S.M.); 6 Unit of Obstetrics and Gynecology, Azienda USL-IRCCS di Reggio Emilia, 42123 Reggio Emilia, Italy; matteo.crotti@ausl.re.it (M.C.); lorenzo.aguzzoli@ausl.re.it (L.A.); vincenzodario.mandato@ausl.re.it (V.D.M.); 7 Epidemiology Unit, Azienda USL-IRCCS di Reggio Emilia, 42123 Reggio Emilia, Italy; lucia.mangone@ausl.re.it; 8 ISSEMYM Hospital, Toluca 50010, Mexico; cbu23bfmc@cbu.ca; 9 Surgical Oncology Unit, Azienda USL-IRCCS di Reggio Emilia, 42123 Reggio Emilia, Italy; maurizio.zizzo@ausl.re.it (M.Z.); andrea.morini@ausl.re.it (A.M.); 10 Department of Medical and Surgical Sciences and Advanced Technologies “G.F. Ingrassia” Anatomic Pathology, University of Catania, 95123 Catania, Italy; giuseppe.broggi@phd.unict.it (G.B.); rosario.caltabiano@unict.it (R.C.); sere.salzano@gmail.com (S.S.); 11 Pathology Unit, Policlinico Foggia, University of Foggia, 71122 Foggia, Italy; francesca.sanguedolce@unifg.it; 12 Second Department of Pathology, Medical School, National and Kapodistrian University of Athens, Attikon University Hospital, 12462 Athens, Greece; nkoufo@med.uoa.gr; 13 Breast Unit, Rea Maternity Hospital, P. Faliro, 17564 Athens, Greece; iboutas@med.uoa.gr; 14 1st Pathology Department, National Medical Research Center for Obstetrics, Gynecology and Perinatology Named After Academician V.I. Kulakov, Ministry of Health of Russia, 117513 Moscow, Russia; a_asaturova@oparina4.ru; 15 Medical Oncology Unit, Azienda USL-IRCCS di Reggio Emilia, 42123 Reggio Emilia, Italy; 16 Radiopharmaceutical Chemistry Section, Nuclear Medicine Unit, Azienda USL-IRCCS di Reggio Emilia, 42123 Reggio Emilia, Italy; sara.rubagotti@ausl.re.it

**Keywords:** episiotomy, pregnancy, squamous cell carcinoma, adenocarcinoma, metastasis, cervical cancer, cervix, vulva

## Abstract

**Background/Objectives**: Rarely, primary (PriCs) or metastatic (metECs) carcinomas occur in the episiotomy site. **Methods**: A systematic literature review of metECs was carried out. We reviewed the PRISMA guidelines and the Scopus, Pubmed, and Web of Science databases. **Results**: We found 21 carcinomas; all of them were cervical carcinomas (11 squamous, SCC; 6 adenocarcinomas; 3 adenosquamous; 1 SCC or adenocarcinoma) diagnosed during pregnancy (38%) or 0.25–8 months postpartum (57%). SCCs were larger (mean size: 4.8 cm). At presentation, only two cases were pN+, and no distant metastases were found, excluding four episiotomy metastases (one anticipating the cervical cancer diagnosis); the remaining episiotomy metastases (mean size: 3 cm; one multifocal) were found at follow-up (these were first metastases in 86% of cases). The time range from the episiotomy/last delivery to first episiotomy metastasis was 1–66 (mean, 12.3) months. Treatment was variable: hysterectomy (71%) ± lymphadenectomy (67%) and/or adjuvant treatment (19%); chemoradiation/radiotherapy alone (24%). A total of 90% of cases recurred after 18 days to 66 months (mean, 12 months). At last follow-up, ten patients (48%) were disease-free after 12–120 (mean, 63.5) months, two patients (10%) were alive with disease, and nine (42%) patients died of disease after 6–36 (mean, 12.5) months (including two never-cleared/progressing cases). **Conclusions**: PriCs and metECs are rare. Iatrogenic/obstetric implantation or vascular dissemination of cervical cancer at the site of episiotomy may occur. For episiotomy lesions, accurate gynecological/perineal examination is required, and biopsy can be considered. Larger studies are required in order to determine treatment guidelines. Compared to PriCs, metECs occurred in younger (premenopausal) patients, were not associated with endometriosis, and demonstrated slightly smaller size and shorter mean time from episiotomy to episiotomy metastases, with a higher likelihood of a less favorable prognosis.

## Simple Summary

Episiotomy is a surgical perineal incision enlarging the vaginal opening during labor to prevent severe perineal lacerations. We previously reviewed the characteristics of primary carcinomas arising from the episiotomy scar. In the present systematic literature review, we analyzed the clinical–pathological features of patients with secondary carcinomas metastasizing to the episiotomy scar site.

## 1. Introduction

Episiotomy is a perineal surgical incision expanding the vagina during the second stage of labor or hysterectomy of a large uterus in order to avoid severe (third–fourth degree) perineal lacerations [1,2,3,4,5,6,7,8,9,10,11,12,13,14,15,16,17,18,19,20,21,22,23,24,25,26,27,28,29,30,31,32,33]. Indeed, about 90% of pregnancies are complicated by perineal traumas, sometimes causing short- and/or long-term morbidities with medicolegal implications and impact on healthcare costs (pelvic floor disorders, dyspareunia, persistent pain, obstetrical anal sphincter injury with incontinence, a negative influence on mother’s ability to care for children) [34,35,36,37,38,39,40,41]. Primiparity, advanced maternal age, Asian ethnicity, operative vaginal birth, prolonged second stage of labor, maternal birth position, fetal malposition (occiput posterior), increased fetal birthweight, type of laceration, repair techniques and materials, and birth assistant’s poor expertise all influence the degree/extent of peritoneal traumas (first degree: only perineal skin injury; second degree: involvement of perineal muscles; third degree: anal sphincter muscle complex injury; fourth degree: extension to rectal mucosa) [34,35,36,37,38,39,40,41,42,43,44]. Episiotomy is widely used, although with wide variation between countries (9.7–100%). However, according to the American College of Obstetricians and Gynecologists (ACOG) and the World Health Organization (WHO), evidence-based data seem insufficient to recommend episiotomy in routine practice; some types of episiotomy may cause adverse consequences such as insufficient prevention or increase in obstetric sphincter ani muscle injuries and hemorrhage [1,2,3,4,5,45,46,47,48,49,50]. Clinical judgment is the best guide; mediolateral episiotomy may have a lower risk of anal sphincter injury (compared to midline episiotomy) when associated with forceps or vacuum delivery but may increase the chances of long-term perineal pain and dyspareunia [45,46,47,48,49,50].

Hormonal and immunologic changes in pregnancy may promote tumorigenesis [51,52,53,54,55]. We previously reviewed the primary malignant carcinomas arising from the episiotomy site (PriCs) [6,7,8,9,10,11,12,13,14,15,16,17,18,56]. We have now performed a systematic literature review to describe the clinical–pathologic features of secondary malignant tumors relapsing/metastasizing to the episiotomy scar site (metECs).

## 2. Materials and Methods

To identify metECs, we conducted a retrospective observational systematic literature review according to the PRISMA guidelines (http://www.prisma-statement.org/; accessed on 4 June 2025) and the PICO process (population: human patients with metECs; intervention: any; comparison: none; outcomes: clinical outcomes (status at last follow-up, and survival and recurrence rates)). We searched for (carcinoma OR carcinomas OR adenocarcinoma OR adenocarcinomas OR cancer OR sarcoma OR sarcomas OR melanoma OR melanomas OR “gestational trophoblastic” OR choriocarcinoma OR choriocarcinomas OR mole OR molar OR “epithelioid trophoblastic” OR “placental site tumor” OR “placental site tumors” OR “placental-site tumor” OR “placental-site tumors” OR “placental site trophoblastic tumor” OR “placental site trophoblastic tumors” OR “placental-site trophoblastic tumor” OR “placental-site trophoblastic tumors” OR “placental site nodule” OR “placental site nodules” OR “placental-site nodule” OR “placental-site nodules” OR “placental site trophoblastic nodule” OR “placental site trophoblastic nodules” OR “placental-site trophoblastic nodule” OR “placental-site trophoblastic nodules”) AND (episiotomies OR episiotomy) in Pubmed (all fields, 112 results; https://pubmed.ncbi.nlm.nih.gov, accessed on 4 June 2025), Scopus (title/abstract/keywords, 148 results; https://www.scopus.com/home.uri, accessed on 4 June 2025), and Web of Science (all fields, 89 results; https://www.webofknowledge.com/, accessed on 4 June 2025) databases. No limitations were set. The bibliographic research ended on 4 June 2025. We applied the following:

- Eligibility/inclusion criteria: studies describing metECs.
- Exclusion criteria: primary episiotomy tumors; tumors not metastasizing to the episiotomy site; unclear diagnosis; results not analyzable (too aggregated or scant data).

**Figure 1:**
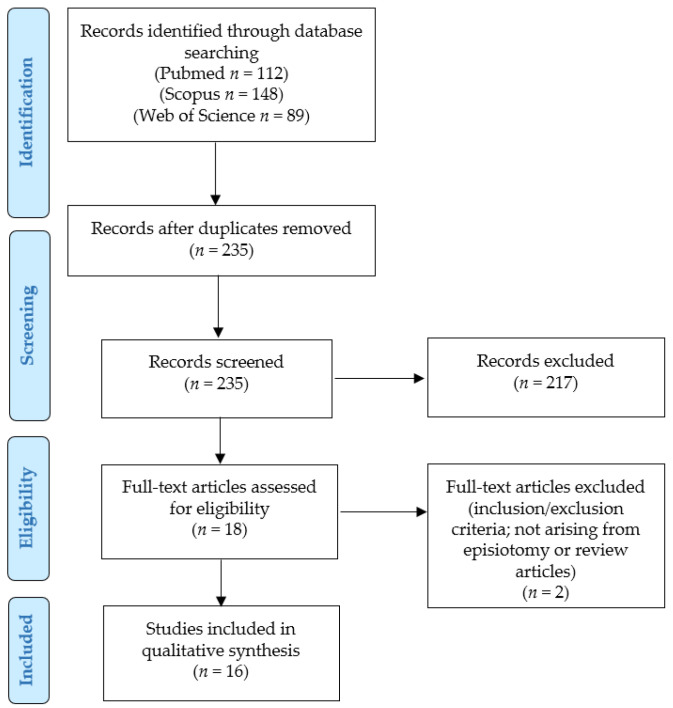
PRISMA flowchart of our systematic literature review.

Two authors independently read the titles and abstracts of all the retrieved results (*n* = 235 after removal of duplicates). Applying the eligibility/inclusion and exclusion criteria, 18 articles were eligible and retrieved as full texts; their reference lists were screened for additional relevant papers. Sixteen articles were finally included after excluding two papers that did not describe additional cases (Figure 1) [17,19,20,21,22,23,24,25,26,27,28,29,30,31,32,33].

Data collection was study- and case-related. We used the R-4.1.3 software (R Foundation, Vienna, Austria) for statistical analysis. Continuous variables were analyzed by ranges and mean values, and their distribution was analyzed using the Shapiro test. Categorical variables were analyzed as frequencies and percentages. Kruskal–Wallis and Fisher’s exact tests were used to assess associations between clinical–pathologic parameters for continuous and categorical variables, respectively. The overall (OS) and recurrence free survivals (RFSs) were counted as the time from surgery to patient’s death/last follow-up (OS) and to recurrence/last follow-up (RFS), respectively. Survival analysis was performed applying by log-rank test. Associations were statistically significant for *p*-values < 0.05.

## 3. Results

### 3.1. Case Series: Diagnosis

We identified 21 metECs, all primarily arising from the uterine cervix [17,19,20,21,22,23,24,25,26,27,28,29,30,31,32,33]. Most studies reported only case reports or small series (<5 patients) [17,29,32]. Unlike PriCs [18], most metECs were diagnosed in the United States (*n* = 10) [21,24,25,26,29,31,32], followed by Europe (*n* = 8; five in the United Kingdom [17,29,33], one in Portugal [20], one in Poland [27], and one in Denmark [22]) and Asia (*n* = 3; Iran [19], Turkey [23], and Lebanon [30]).

The 21 metECs included eleven squamous cell carcinomas (SCCs) [17,23,25,27,29,30,31,33] (one grade 1 [27], one grade 2 with minimal stromal invasion [25], eight grade 3 [17,19,20,21,22,23,24,26,28,29,30,32,33], one unclear grade [31]), six endocervical adenocarcinomas (ADCs) [17,19,24,28,32] (one grade 1 [24], two grade 2 [19,32], three grade 3 [28,32]), three adenosquamous carcinomas (ASCs) [20,21,22] (one grade 2 [21], two grade 3 [20,23], including a glassy cell carcinoma [20]), and a case with an unreported histotype (SCCs vs. ADCs) [26] (Table 1).

Two ADCs showed histological patterns of human papillomavirus (HPV)-related E- ADC (one usual type, HPV16+ [19], one villoglandular [24]); the remaining cases seemed to be more HPV-associated and/or showed HPV-related precursors (in situ adenocarcinoma, AIS; high-grade intraepithelial lesion/cervical intraepithelial neoplasia, H-SIL/CIN3) [24,28,32,33]. Immunohistochemistry was performed on only one ADC (EMA+, CK7+, CDX-2+ weak, CK20-) [19]. Molecular analysis was not performed in any case.

### 3.2. Cervical Cancer Presentation

Patients were all premenopausal (age range: 21–38 years; mean age: 31.75 years; median age: 32 years) [17,19,20,21,22,23,24,25,27,28,29,30,31,32,33] and mostly multiparous (*n* = 12, 75%) [17,21,22,23,24,25,27,28,30,31,32,33]. The mean (34.7 years) and median age (35 years) were slightly higher for ASCs (range, 34–35 years) [20,21,22] than ADCs (range, 29–38; mean, 32.2; median, 32 years) [17,19,24,28,32] or SCCs (range, 21–37; mean, 30.7; median, 32 years) [17,23,25,27,29,30,31,33], but few cases were described (Table 1).

All the cases of cervical cancer (CC) seemed to be pregnancy-associated according to some authors’ criteria (diagnosis during pregnancy or within a year postpartum) [17,19,20,21,22,23,24,25,26,27,28,29,30,31,33,57,58,59]. CCs were histologically diagnosed at the time of delivery in eight (38%) cases [21,22,24,29,31,32] or 0.25–8 (mean, 3) months postpartum in 12/21 (57%) cases [17,19,20,23,25,27,28,29,30,32,33] (one unclear [26]), but the CCs clearly presented during pregnancy in 4/20 cases [19,24,29,32], and onset during pregnancy was usually not excluded (Table 2).

Data about Pap smears were available for 14 patients (Table 1) [17,19,20,22,24,25,27,28,29,31,32,33]. Six (43%) Pap tests were positive, including three SCCs [17,29,33], two ADCs [19,24], and one ASC [20]. A total of 4/6 (67%) smears were performed 1.5–7 (mean, 3.9) months postpartum [17,19,20,33], while in two cases, the cytological diagnosis was achieved prenatally [24,29]. Two (14%) additional prenatal Pap smears showed atypical findings insufficient for a dysplastic or neoplastic diagnosis [22,28].

Other diagnostic procedures included one dilation and curettage [19], twelve biopsies [17,20,23,25,28,29,30,31,32,33], three excisions/polypectomies [17,22,24], and two cone biopsies [28,33].

CCs usually presented as exophytic [20,22,23,27,30], cauliflower-like [33], mass [31], pedunculated/raised/polypoid [17,19,24,25,27,33], and eroded/bleeding/ulcerated lesions [27,32,33]. The tumor involved the anterior (four cases) [22,24,28,33], antero-inferior (one case) [19], or posterior cervix (three cases) [17,31,32], completely replacing the exocervix [25] or also involving the vaginal posterior upper third [17] in one case each.

The CC size range was 0.5–7 cm (mean, 2.9; median, 2 cm) (*n* = 12) [17,19,20,22,23,24,25,28,31,32]. The mean (4.8 cm) and median (5 cm) sizes were larger for SCCs (range, 2–7 cm) [17,23,25,31] than ADCs (mean/median: 1.3 cm; range: 0.5–1.8 cm) [17,19,24,28,32], but few data were available. The information regarding only two ASCs was reported (3 and 5 cm, respectively) [20,22] (Table 3).

Bloody vaginal discharge/spotting (six cases) [17,19,23,24,27,30] sometimes also occurred in a post-coital setting (three cases) [17,23,30]. Three patients complained of dyspareunia and/or pelvic/flank/perineal pain [23,30,33]. Symptoms lasted 1.5–24 (mean, 9) months [17,19,23,30]. Three patients were asymptomatic [17,22,28] (Table 3). Serum tumor markers levels were unavailable. Imaging data were too scant; computed tomography scans seemed to find the episiotomy metastasis in 1/5 cases [17,22,23,25,27].

When reported, all the patients delivered through their vagina [17,19,20,21,22,23,24,25,27,28,29,30,31,32,33]; no cesarean sections were clearly reported. Data on the local conditions at the time of admission for delivery were scant. Excluding CC presentation, other complications included: one postpartum hemorrhage due to transection of polypoid ADC during episiotomy [24]; one delivery of a piece of tumor with the infant [22]; one isthmo-cervical insufficiency at the 25th gestational week, preterm rupture of membranes and delivery at 32 gestational weeks, rupture of the cervix during labor, and prolonged blood-like vaginal discharge during postpartum [27]. When reported, all the babies were healthy [22,24,25,27,28,33], and episiotomy was midline (*n* = 7, 33%) [20,24,25,30,31,32] (one second degree [24]) or mediolateral (*n* = 2, 10%) [22,23]. Episiotomy, episiorrhaphy, excochleation of the uterus, and suturing of the cervix were performed in one case [27]. Kielland’s forceps were used in one case [33].

### 3.3. Tumor Stage and Primary Treatment

As to the FIGO stage classification [60,61], two cases were stage IA1 [24,28], nine were IB (three were not otherwise specified [21,29,33], five were IB1 [17,19,31,32], one was IB3 [22]), one was IIA [26], one was IIIB [30], and two were IIIC1 (pN+) [29]. In the other cases, the stage was unclear, including four cases with episiotomy metastases at presentation (IIIa vs. IV) [17,20,23,27] (Table 3). The parametria and vaginal posterior upper third were invaded in 4/21 (19%, reaching the pelvic side wall in one case) [17,23,26,30] and 1 case [17], respectively.

Lymph node enlargement was identified in 1/5 (25%) cases [17,19,20,27,30], lymphovascular invasion in 2/6 (33%) pN0/pNx primary hysterectomy specimens [19,20,22,24,25,32]. Perineural invasion was absent in 3/3 cases [19,20,24].

Extra-episiotomy distant metastases were not clearly reported at presentation, except possibly for a case with unclear timing of lung metastases (presentation vs. recurrence) [29].

The time from CC diagnosis to primary treatment ranged from 11 days to 4.5 months (mean, 1.8 months) [17,31,33]. The time from delivery to primary treatment ranged from 11 days to 6 months (mean, 3.3 months) [17,20,22,24,27,28,31,33] (Table 2).

Radical/total abdominal hysterectomy was performed in 15 (71%) patients [17,19,20,21,22,24,25,28,29,31,32], with additional bilateral salpingo-oophorectomy (2 cases) [17,32], salpingectomy (1 case) [19], pelvic (14 cases, 67%) [17,19,20,22,24,25,28,29,31,32] or pelvic/para-aortic lymphadenectomy (2 cases) (Table 3) [24,25]. The surgical margins were free of tumor in six cases [17,19,20,22,25,31] and positive for AIS/CIN3 in one cone biopsy [28] or SCC in another case [33], but there was no residual disease in the hysterectomy specimen [28] or after radiotherapy (RT) [33], respectively. Adjuvant treatment was administered to four (19%) women, including chemoradiation (two cases) [17,22] or RT (two cases) [21,33], both with [22,33] or without brachytherapy (BT) [17,21]. Five (24%) patients underwent exclusive chemoradiation (two cases) [23,27] or RT (three cases) [26,30,33]. Globally, BT was added to external RT in four (19%) cases [17,27,30,33].

### 3.4. Episiotomy Metastases, Tumor Recurrences, and Follow-Up

In four (19%) cases, the episiotomy metastases were found at presentation, synchronously with (three cases) [17,20,23] or 1 month before (one case) [27] the CC presentation. Surgical excision of the nodules was performed in 2/4 (50%) cases with or without ChT/RT/BT [17,20], while in the remaining cases, chemoradiation (±BT) was administered without surgery [23,27]. Two of four cases did not recur [17,23], one relapsed once at the perineum [20], and one recurred twice at the episiotomy/perineal/vulvar site [27]. In the remaining 17 (81%) patients, the episiotomy metastases presented as a single (*n* = 15, 71%) [17,19,21,24,25,26,28,29,30,31,32,33] or double recurrence (*n* = 2, 10%) [22,32] during follow-up [17,19,20,21,22,24,25,28,29,30,31,32,33]. Globally, 19/21 (90%) cases recurred [17,19,20,21,22,24,25,26,27,28,29,30,31,32,33], and episiotomy recurrences were found in 18 (86%) patients [17,19,20,21,22,24,25,27,28,29,30,31,32,33].

Excluding cases never cleared from disease (unstoppable disease progression) [20,27], the time to first recurrence ranged from 18 days to 66 months (mean, 12 months) (Table 2 and Table 3) [17,19,20,21,22,24,25,26,27,28,29,30,31,32,33]. The mean time from episiotomy or last delivery to the first episiotomy metastasis was 12 months, although the range was wider (1–66 months) [17,19,20,21,22,23,24,25,26,27,28,29,30,31,33,57,58,59]. The time from the first to second recurrence ranged from 6 weeks to 3.25 months (mean, 2.4 months) [20,22,24,28,32]. Data of recurrences after the second relapse were unclear.

The first metastatic site at presentation/recurrence was local (episiotomy and nearby tissues) in most patients (*n* = 18, 86%) [17,19,20,21,22,23,24,25,26,27,28,30,31,32,33] or regional (pelvic lymph nodes) in two (10%) cases [29], while in one case, it was unclear if lung metastases occurred at presentation or recurrence [29]. Non-local (regional/distant) metastases were more frequently subsequently involved at follow-up (*n* = 5, 24%) (four inguinal lymph nodes, 19% [20,22,28,32]; one left obturator lymph node [24]; one para-aortic lymph nodes, liver, left lung base) [28]; one widespread intra-abdominal and sigmoid colon [22].

The episiotomy metastases were described as swelling [28], mass/lump [19,29,31,33], granulation tissue-like [31], nodular [17,20,22,25], cystic [32], nodular/cystic, discharging clear mucinous material [24], polypoid [32], ulcerative [23,27], firm/hard [20,24,30], and/or necrotic [30]. Pain (three cases) [19,22,27], vaginal/pelvic discharge (three cases) [22,24,30], and dyspareunia (one case) [30] were rarely reported. The mean episiotomy tumor size was 3 cm (range, 0.5–6.0 cm) (Table 4) [17,19,20,22,23,24,25,27,28,30,31,32,33]. In one case, two synchronous episiotomy lesions were identified [22]. The following sites were involved: vulva/perivulvar soft tissues [20,22,30,31], vagina (lower third) [19,23,27], perineum [19,20,21,24,27,32], pelvis [24,29,32], recto-vaginal septum [25,32], perirectal fat/external anal sphincter [19,33].

In six cases, the episiotomy lesion was misdiagnosed as a benign condition (one inflammation [19], three abscesses [27,29], two epithelial inclusion cysts [29,32]), usually causing diagnostic/treatment delay (range: 2–9 months; mean, 6 months) [27,29,32]; indeed, 3/6 patients first underwent medical treatment or follow-up before diagnostic biopsies [17,27,29].

Diagnostic procedures for recurrences included not only incisional tumor and/or lymph node biopsies but also fine-needle aspiration cytology (FNAC); FNACs were positive in 1/2 (50%) of the episiotomy tumors [24,32] (negative case: granulation tissue and benign columnar cells, epithelial inclusion cyst) [32] and in 2/3 (67%) of inguinal lymph nodes [20,27,28].

In terms of the treatment of recurrences, surgery was carried on in 12/19 (63%) cases [17,21,22,25,28,29,31,32,33] as the exclusive treatment (two alone, 11%) [32,33], after RT (one case, 5%) [29], or followed by RT and/or BT (six cases, 31%) [17,25,28,29,31,32] or chemoradiation/BT (one case, 5%) [22]; different schedules were also reported (two cases, 11%) (Table 4) [21,29].

The histopathological exam of the episiotomy nodules (either at presentation or at recurrence) showed disease-free surgical margins after treatment in seven cases [17,20,21,24,28,31,32] (in one case, lymphovascular invasion was close to the surgical margins) [28]; data were unclear in the remaining cases undergoing excision.

A total of 7/19 (7%) patients were treated with exclusive chemoradiation (three cases) [19,20,30], RT (three cases) [24,26,27], or chemotherapy (ChT) (one case) [29]. Globally, ChT was administered in eight (38%) cases [19,20,21,22,28,29,30]; RT in 15 (79%) cases (total dose: 14–69 Gy, mean, 43 Gy; 7–28 fractions, mean, 23) [17,19,20,21,22,24,25,26,27,29,30,31,32], typically irradiating the whole pelvis/perineum, sometimes extended to the lomboaortic [19] or inguinal lymph nodes [24]; and BT was administered in seven (37%) cases (total dose: 20–63.8 Gy, mean, 32.5 Gy) [17,19,22,25,28,31,32].

The five (24%) patients with details about the second recurrence were treated by surgery alone (one case) [24], exclusive ChT (one case) [20], ChT + surgery (one case) [22], chemoradiation (one case) [28], or surgery + RT (one case) [32].

The best response to therapy was complete in ten (47%) cases [17,19,21,23,24,25,28,31,32], partial in four (19% of cases [17,22,30,33]), or unknown in five (24%) cases [26,29], while there was no response with disease progression (PD) in two (10%) cases [20,27].

Side effects of recurrence treatment (six cases, 32%) were described after chemoradiation (two cases) [21,27], RT (three cases) [25,31,32], or ChT (one case) [28], typically being local/pelvic (five cases) [21,25,27,31,32] and requiring additional surgery in three cases [21,31,32]; more systemic toxicity was found in two patients [27,28] (Table 4).

Follow-up data (Table 3) were available for 21 patients (range, 6–120; mean, 40 months) [17,19,20,21,22,23,24,25,26,27,28,29,30,31,32,33]. Ten patients (48%) were disease-free 12–120 (mean 63.5) months after diagnosis [17,19,21,23,24,25,29,31,32], two (10%) were alive with disease at the last clinical exam [17,33], and nine (42%) were dead of disease 6–36 (mean, 12.5) months after diagnosis [20,22,26,27,28,29,30].

### 3.5. Statistical Analysis

Mean age, tumor histological grade, CC size, parametrial involvement, FIGO stage, pN stage, CC presentation during pregnancy vs. postpartum, presence/absence of episiotomy metastasis at presentation or recurrence (globally, and single vs. double recurrence), time from delivery to episiotomy tumor, type of treatment of primary tumor and recurrences (surgery, lymphadenectomy, RT, BT, ChT, and combinations of such treatments), and best response to therapy were analyzed to search for potential associations with histotype (SCC, ADC, ASC), OS, and RFS.

As expected, a statistical difference was found among different tumor stages (*p* = 0.025). The mean CC size was significantly smaller (*p* = 0.019) for ADCs (1.3 cm) [17,19,24,28,32] than ASCs (4 cm) [20,22] or SCCs (4.8 cm) [17,23,25,31], but data were based on just a few cases. No statistical significance was identified (*p* < 0.05) for all the other analyzed variables due to the scant data and low number of reported cases.

## 4. Discussion

### 4.1. Cervical Cancer and Pregnancy: Overview and Risk Factors

We found only 21 metECs [17,19,20,21,22,23,24,25,26,27,28,29,30,31,32,33] and 13 PriCs [6,7,8,9,10,11,12,13,14,15,16,17,18,56]; non-carcinomatous malignancies (melanomas, sarcomas, or trophoblastic tumors) arising from other gynecological or extragynecological sites were not identified in both groups [56,61,62,63,64,65,66,67,68,69,70,71,72,73,74,75,76,77,78]. All metECs arose from the uterine cervix and seemed pregnancy-related [17,19,20,21,22,23,24,25,26,27,28,29,30,31,32,33]. CC is the fourth most common female cancer and leading cause of death in low-income countries particularly (SEER data: 13,820 new cases and 4360 new deaths in 2024; 5-year OS: 67–77%) [79,80,81,82,83,84].

Different tumors have been variably associated with pregnancy (breast, gynecological, salivary gland, adrenal, germ cell, lymphomas, melanoma, etc.); for breast cancer, pregnancy is either a risk factor (even 5 years postpartum; hormone receptor-negative cases; age > 35 years) or protective event (early pregnancies, age < 25 years) [22,23,24,25,26,52,57,58,59,85,86,87,88,89,90,91,92,93,94,95,96,97,98,99,100,101,102,103,104,105,106,107,108,109,110,111,112,113,114,115,116,117,118,119,120,121,122,123,124,125,126,127,128,129,130,131,132,133,134,135,136,137,138,139,140,141,142,143,144,145,146,147,148,149,150,151,152,153,154,155,156,157,158,159,160,161,162,163,164,165,166,167,168,169,170,171,172,173,174,175,176,177,178,179,180,181,182,183,184,185,186,187]. After breast cancer, CC is also the most frequent pregnancy-associated carcinoma (0.1–12:10,000 pregnancies; 0.05–3% of pregnant/postpartum women) among gynecologic cancers (72%); its incidence varies as to: age; socioeconomic conditions; patients’ choices; type of medical care, prenatal testing, and screening during pregnancy; and inclusion of cases diagnosed during pregnancy and postpartum [57,58,59,85,86,87,88,89,90,91,92,93,94,95,96,185,186,187,188,189,190,191].

CCs diagnosed several months after delivery may have a weaker link to pregnancy, while most (80%) CCs diagnosed during pregnancy seemed to be at the early stage, allowing gestation continuation and/or fertility-sparing approaches with higher chances of complete remission [85,87,88,89,90,91,92,192,193,194,195]. Moreover, CCs at the early stage may be underdiagnosed in pregnancy, as vaginal bleeding/discharge is also due to non-oncologic complications, and colposcopic abnormalities can be difficult to detect due to increased mucus production, cervical hyperemia, gland prominence, and columnar epithelium eversion [196,197,198,199,200,201,202,203,204]; most (57%) CCs were diagnosed postpartum (even after episiotomy metastasis), although a clinical lesion and/or an abnormal Pap smear were sometimes found during gestation. In any case, large series of pregnant CC patients were lacking; pregnant and non-pregnant cases showed no prognostic difference in most studies if compared as age- and stage-matched groups. However, some CC series diagnosed during late pregnancy or postpartum showed worse prognosis and recurrence risk, while the prognostic difference between vaginal and cesarean delivery is controversial [26,29,57,58,59,85,205,206,207,208,209,210,211,212,213,214,215,216,217,218,219].

Risk factors for CC include HPV and other sexually transmitted infections, age at first intercourse, number of sexual partners, smoking, multiparity, and immunodepression; the role of obesity is unclear, with less compliance to screening, impact on pregnancy outcomes, more comorbities/postoperative complications, and difficulty managing anesthesia and surgery but a possible protective effect against chemoradiation [220,221,222,223,224,225,226,227,228,229,230,231,232,233,234,235,236,237]. Our series showed scant data.

**Figure 2:**
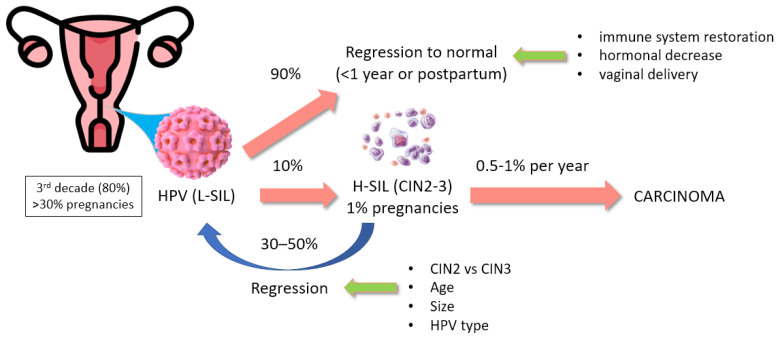
HPV/L-SIL progression to H-SIL/carcinoma.

HPV infection (LSIL) is more frequent in young adults (80% at the third decade), pregnant women (>30%), and multiparous women, while high viral loads seem associated with negative pregnancy outcomes; most (90%) LSILs regress within 1 year or postpartum (>80%) due to immune system restoration, hormonal decrease, and vaginal delivery (60–66% vs. 12% of cesarean sections), which causes cervical trauma/inflammation, repair, and/or transient ischemia [80,210,223,238,239,240,241,242,243,244,245,246,247,248,249,250,251,252,253,254,255,256,257,258,259,260,261,262,263,264]. Globally, 10% of LSILs progress to HSIL, which can either regress to normal/LSIL (30–50%; especially CIN2, depending on age, size, and HPV type) or progress to cancer (0.5–1% cases per year). HSIL is diagnosed in ~1% of pregnant patients and persists/progresses postpartum in 2–8% of cases (Figure 2) [80,210,223,238,239,240,241,242,243,244,245,246,247,248,249,250,251,252,253,254,255,256,257,258,259,260,261,262,263,264].

Almost 90–95% CCs and precursors are HPV-related. In situ hybridization (using probes labeled with radioisotopes or chemically reactive ligands highlighted by autoradiography, fluorescence, or color reaction) or molecular analysis (such as polymerase chain reaction analysis/genotyping assays) could be performed on tissue or liquid biopsies to detect HPV infection and identify the HPV subtype/risk category. Low-risk HPV types (such as 6, 11, 42, 43, and 44) are typical of low-risk SIL (L-SIL) and only occasionally found in CC, while high-risk types (such as 16, 18, 31, 33, 34, 35, 39, 45, 51, 52, 56, 58, 59, 66, 68, and 70) are more typical of H-SIL/CC; intermediate risk HPV types also exist [265,266].

p16 (not tested in our cases) is an immunohistochemical surrogate for HPV (tested in only one metEC) but it can be also overexpressed in HPV-independent gynecological or extragynecological cancers (such as high-grade serous, urothelial carcinomas, etc.) [51,80,184,238,267,268,269,270,271,272,273,274,275,276,277,278,279,280,281,282,283,284,285,286,287,288,289,290,291,292,293,294,295,296,297,298,299,300,301,302,303,304,305,306,307,308,309,310]. A new histological pattern-based prognostic classification of HPV-related cervical ADCs was recently proposed (unavailable data in our series) [80,311,312,313,314,315]; HPV-independent ADCs (mesonephric, gastrointestinal-type, etc.) [51,80,184,267,268,269,270,271,272,273,274,275,276,277,278] are rarer and aggressive (not found in our series).

### 4.2. Cervical Cancer and Pregnancy: Possible Pathogenic Factors

**Figure 3:**
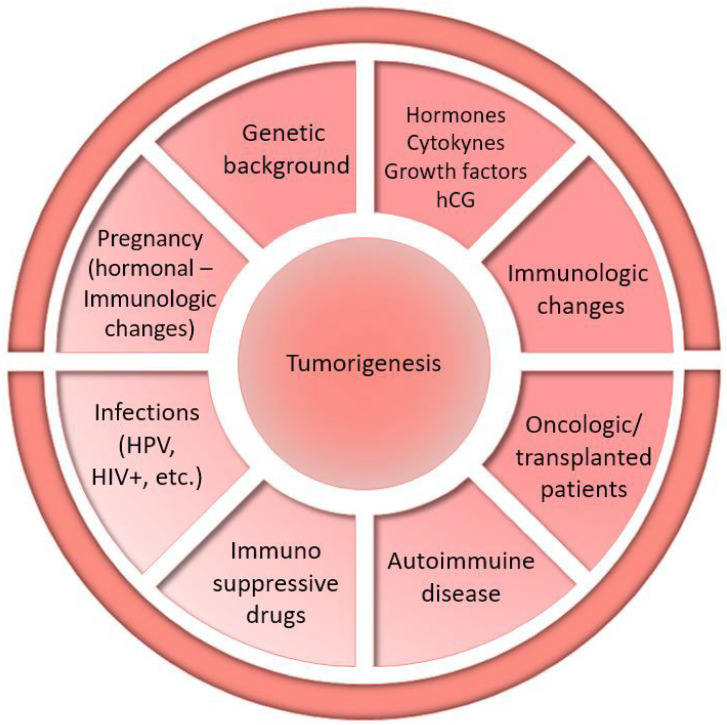
Factors and co-factors involved in tumorigenesis.

Immunodepression (oncologic or transplanted patients, HIV+, autoimmune diseases, or immunosuppressive drugs) promotes HPV and other infections, precancerous lesions, and solid and hematologic neoplasms; the hormonal and immunologic changes in pregnancy may also favor the genesis and progression of different tumors, sometimes expressing hormone receptors or human chorionic gonadotropin (hCG) and stimulated by sex hormones and growth factors (Figure 3) [22,23,24,25,26,51,52,53,54,55,57,58,59,85,86,87,88,89,90,91,92,93,94,95,96,97,98,99,100,101,102,103,104,105,106,107,108,109,110,111,112,113,114,115,116,117,118,119,120,121,122,123,124,125,126,127,128,129,130,131,132,133,134,135,136,137,138,139,140,141,142,143,144,145,146,147,148,149,150,151,152,153,154,155,156,157,158,159,160,161,162,163,164,165,166,167,168,169,170,171,172,173,174,175,176,177,178,179,180,181,182,183,184,254,255,256,257,264,316,317,318,319,320,321,322,323,324,325,326,327,328,329,330,331,332,333,334,335,336,337,338].

Indeed, pregnancy-associated transient immune suppression/modulation allows tolerance to the fetus but reduces immune control against infections favoring HPV DNA integration and CC carcinogenesis and progression; fetomaternal microchimerism can also modulate the risk for maternal cancer [80,206,240,241,242,243,244,245,246,264,339,340,341,342].

In pregnancy, hormones, cytokines, and other factors alter the cervical immune microenvironment; for example, TGF-β reduces the maturation/recruitment of cervical dendritic antigen-presenting cells. Moreover, increased estrogen and progesterone levels decrease GM-CSF expression, promote proliferation and differentiation of the cervical epithelium, and induce cervical hypertrophy/congestion with persistent external exposure of the cervical transformation zone and susceptibility of metaplastic cervical epithelium to HPV (also due to pregnancy-related vaginal flora imbalance due to a humidified microenvironment); they may enhance viral DNA transcription, persistence/replication, and integration in the host genome, with risk for carcinogenesis [59,85,86,87,88,89,90,91,92,93,94,95,96,138,181,182,183,184,205,264,316,317,318,319,320,321,322,323,324,325,326]

hCG (mainly produced by the syncytiotrophoblast) seems positively correlated with HPV infection; it interacts with hCG/LH receptor (expressed by endothelial cells) and induces VEGF expression in macrophages, causing a neoangiogenic effect [343,344,345,346,347,348,349,350,351,352,353,354,355,356,357,358,359,360,361,362,363,364,365,366,367,368,369,370,371,372,373]. Neoangiogenesis, increased permeability, and vasodilation in the female genital tract promote the development of the placenta and proper fetomaternal circulation (otherwise dysregulated in gestational diabetes mellitus, preeclampsia, fetal growth restriction, or vascular anomalies such as hemangiomas or arteriovenous malformations); these changes may favor CC vascular dissemination, especially after vaginal delivery [9,10,11,43,192,205,264,374,375,376,377,378,379,380,381,382,383,384,385,386,387,388]. The physiological pregnancy-associated remodeling of uterine cervix and fetal membranes due to an increased size of the fetus and uterus can be due to increased matrix metalloproteinases (MMPs) activity, not necessarily linked to neoangiogenesis [389,390,391,392]. However, MMPs overexpression causes loss of cellular adhesion, extracellular matrix remodeling, and neoangiogenesis in cancer models, promoting epithelial–mesenchymal transition, tumor cell proliferation, infiltration/progression, and vascular dissemination; it is a poor prognostic factor in CC [207,208,393,394,395,396,397,398,399,400,401,402,403,404]. The relationship and effects of pregnancy, CC and MMP activity should be further investigated.

### 4.3. Episiotomy Metastases: Overview, Dissemination Pathways and Prognosis

**Figure 4:**
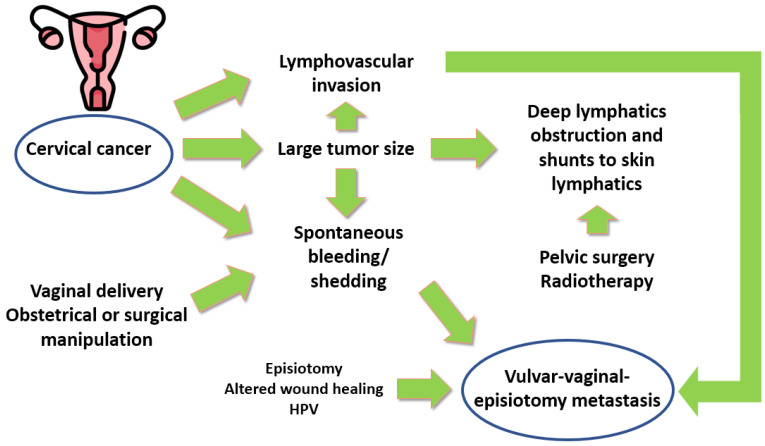
Potential pathways of CC dissemination to the episiotomy.

The vagina is preferentially contaminated by bleeding/shedding of CC or endometrial cancers, but lower vaginal, vulvar, and perineal metastases are unusual (Figure 4) [17,19,20,21,22,23,24,25,26,27,28,29,30,31,32,33,405,406,407,408,409,410,411,412,413,414,415,416,417,418,419,420,421,422,423,424,425,426,427,428,429].

Skin metastasis are very rare in CCs (0.01–2%), typically at advanced stage (5%), rarely at stage I–III (0.8–1.2%); about 5% stage I–II CCs may recur in the vaginal vault while perineal metastases imply a 40% mortality [318,430,431,432,433,434,435,436,437,438].

Assigning the proper stage to episiotomy metastases from CCs could be challenging; it is unclear if the prognosis could be more similar to a stage IIIA (extending to the lower vaginal third) or IV disease (direct spread beyond the true pelvis/distant metastasis) [60,61,439]. First, data about metECs are scant and the exact metastatic site is mostly unclear (internal lower vagina vs. external vulva/perineum).

Second, episiotomy metastases are usually the first metastatic site and some CCs recurred there a couple of times before further dissemination. Episiotomy metastases may even anticipate the CC detection or be identified at presentation (4/21 cases) [17,20,23,27], not necessarily implying an already widespread disease; however, recurrence and progression are currently unpredictable (evident in 2/4 cases, 50%). In the literature, about 11–20% of CCs were pN+ (FIGO stage IIIC, 34% 5-year OS) [60,61,82,83,438,439,440,441,442,443]; similar rates may have been reported in pregnant patients, but few cases were described [80,83,84,90,444,445,446,447,448]. Regional lymph node metastases were evident in 2 metECs at presentation, while clear non-episiotomy distant metastases were not reported. Globally, most (90%) metECs recurred (mean time, 12 months) but almost the same percentages of patients were disease-free (48%) or died of disease [17,19,20,21,22,23,24,25,26,27,28,29,30,31,32,33].

Third, episiotomy metastases due to implant of tumor fragments detached during spontaneous vaginal delivery or iatrogenic (obstetrical or surgical) manipulation may have different prognosis and dissemination potential compared to CCs reaching episiotomy through surgical incisions/needle tracts or widespread lymphovascular dissemination; pneumoperitoneum may also alter the peritoneal surfaces favoring cancer cell adherence, but data are limited [405,406,431,449,450,451,452,453]. Vaginal delivery may not have a significant prognostic impact on asymptomatic, untreated, microscopic CCs/precursors, but it may be complicated by bleeding, sepsis, cervical laceration, dystocia, obstructed labor, and/or CC fragmentation and implant on other sites (especially if wounded, like episiotomy) or lymphovascular dissemination [206,405,448,449,450]. So, cesarean sections are recommended for large/advanced CCs, although this procedure, as for endometriosis, cannot also prevent dissemination though abdominal incisions to groin lymph nodes or other sites [55,454,455,456,457,458,459]. Large/advanced CCs can obstruct the deep lymphatics, shunting the flow to skin lymphatic vessels; the altered flow may promote lymphovascular dissemination to unusual sites. Extensive radical pelvic surgery or RT enhance lymphatic stasis favoring retrograde spread of neoplastic emboli to vulva [405,407,439,448,449,450,451,460,461,462,463,464,465].

Wound metastases were previously reported also in other cancers (endometrium, breast, gastrointestinal tract) [31,407,408,409,410,411,466,467,468,469,470,471,472,473,474,475,476]. Acute inflammation first responds to surgery, attracting immune cells to the wound field and promoting healing by secretion of cytokines and of growth and angiogenic factors and activating sympathetic nervous signaling. Subsequent immunosuppression may favor residual cancer cells’ growth and migration. Episiotomy wound inflammation is usually transient and local, sometimes becoming chronic/persistent, especially in cases of autoimmune disease, endometriosis, or poor healing (potentially favored by HPV infection); rare complications include fistulae (absent in our series) [412,413,414,415,416]. Inflammation may also clinically mask an underlying tumor.

### 4.4. Episiotomy Metastases: Differential Diagnoses

In some cases, misdiagnosis delayed treatment. The clinical–pathologic differential diagnoses of episiotomy nodules/ulcerated lesions include vulvo–vaginal–perineal tumors (benign or malignant primaries, or metastases) and other benign conditions like endometriosis, granulomas, sarcoidosis, granulation tissue, inflammation, or sexually-transmitted genital infections (syphilis, chancroid, lymphogranuloma venerum, HSV, EBV, etc.); in particular, infections should be considered in order to avoid fetal/pregnancy complications (pregnancy loss, preterm delivery/rupture of membranes) [6,7,8,9,10,11,12,13,14,15,16,17,18,19,20,21,22,23,24,25,26,27,28,29,30,31,32,33,56,476,477,478,479,480,481,482,483,484,485,486,487,488,489,490,491,492,493,494,495,496,497,498,499,500,501,502,503,504,505,506,507,508,509,510,511,512,513,514,515,516,517,518,519,520,521,522,523]. EBV (sometimes detectable in the cervix, vagina, urethra, and anus in non-sexually active women as well) may be reactivated in pregnancy, rarely causing vulvar/vaginal ulcers. Some upper aerodigestive or hematologic tumors are EBV-related, while EBV was exceptionally associated with CC/vulvar carcinomas; EBV was not tested in metECs [524,525,526,527,528,529,530,531,532,533,534,535,536,537,538,539,540,541,542,543,544].

Frozen sections (not performed in metECs) help surgeons to obtain provisional diagnoses but are time-consuming for pathologists, implying costs, risk of small specimens’ exhaustion, and freezing histological artifacts interfering with histological interpretation; they should be performed only if their result alters the ongoing surgery [545,546].

Episiotomy nodule biopsies can be helpful. Many gynecological and extragynecological carcinomas (gastrointestinal, genitourinary, breast, etc.) may exceptionally metastasize to uncommon sites such as the vulva, mimicking a primary tumor; accurate clinical–radiological exams are fundamental to identify and stage the primary tumor [482,483,484,485,486,487,488,489,547].

Table 5 summarizes the main features of PriCs and metECs.

As to the CC epidemiology, metEC patients were younger than PriC patients (32 vs. 50–53 years) and all premenopausal (typical CC onset: 35–45 years), while most (61%) PriCs were postmenopausal.

PriCs showed more frequent association with endometriosis (0% of metECs), slightly larger mean size (4.6 vs. 3 cm; range, 1–10 vs. 0.5–6.0 cm), longer mean time from episiotomy to first episiotomy tumor (21 vs. 12 months; range, 3–30 vs. 1–66 years), lower nodal (3/13 cases, 23%) or distant metastases (0%) rates at presentation, and a higher likelihood of more favorable prognosis; indeed, only three (23%) PriCs recurred and 2/3 (15%) of cases progressed and died of disease (vs. 42% of metECs) [6,7,8,9,10,11,12,13,14,15,16,17,18,19,20,21,22,23,24,25,26,27,28,29,30,31,32,33,56]. Most metEC (75%) and endometriosis-independent PriC patients (75%) were multiparous, while 60% of endometriosis-related PriC cases were primiparous [6,7,8,9,10,11,12,13,14,15,16,17,18,56,57,58,59,205,239,315].

The reported PriC histotypes (clear cell, endometrioid, adenoid cystic carcinomas) were usually different from metECs; moreover, clear cell and endometrioid carcinomas more typically occur in the endometrium or ovaries. Other rare gynecological carcinomas (intestinal-type, neuroendocrine, mesonephric, etc.) were not identified in both groups [6,7,8,9,10,11,12,13,14,15,16,17,18,56,548,549,550,551]. Conversely, SCCs at episiotomy sites could be either PriCs or metECs [16,17]; the histopathological and immunohistochemical exams could not be helpful for this differential diagnosis, except, perhaps, in a case where an in situ component is found, favoring a diagnosis of a primary lesion. Unfortunately, HPV infection could be found either in cervical, vulvar, or extragynecological carcinomas (such as urothelial carcinomas, etc.) [265,266,267,268,269,270,271,272,273,274,275,276,277,278,279,280,281,282,283,284,285,286,287,288,289,290,291,292,293,294,295,296,297,298,299,300,301,302,303,304,305,306,307,308,309,310,311,312,313,314,315,316,317,318,319,320,321,322,323,324,325,326,327,328,329,330,331,332,333,334,335,336,337,338,339,340,341,342,343,344,345,346,347,348,349,350,351,352,353,354,355,356,357,358,359,360,361,362,363,364,365,366,367,368,369,370,371,372,373,374,375,376,377,378,379,380,381,382,383,384,385,386,387,388,389,390,391,392,393,394,395,396,397,398,399,400,401,402,403,404,405,406,407,408,409,410,411,412,413,414,415,416,417,418,419,420,421,422,423,424,425,426,427,428,429,430,431,432,433,434,435,436,437,438,439,440,441,442,443,444,445,446,447,448,449,450,451,452,453,454,455,456,457,458,459,460,461,462,463,464,465,466,467,468,469,470,471,472,473,474,475,476,477,478,479,480,481,482,483,484,485,486,487,488,489,490,491,492,493,494,495,496,497,498,499,500,501,502,503,504,505,506,507,508,509,510,511,512,513,514,515,516,517,518,519,520,521,522,523,524,525,526,527,528,529,530,531,532,533,534,535,536,537,538,539,540,541,542,543,544,545,546,547,548,549,550,551,552]. Only two PriCs (clear cell carcinomas) [6,7] were previously tested by molecular analysis searching for DNA mutations, without significant results, while no metECs were tested; further studies are required. For all these reasons, clinical information is fundamental and should be provided to the pathologists in order to make the proper diagnosis.

Finally, non-recognized, pre-existing, early-stage, pregnancy-associated vulvar carcinomas may be cut by the episiotomy procedure [51], or vulvar tumors may exceptionally attract other tumor implants (tumor-to-tumor metastasis) [448,463]. No metECs were implanted on a PriCs, according to our review.

### 4.5. Strength and Limits of Our Study

We performed a multidisciplinary systematic literature review of an infrequently analyzed topic, screening multiple databases. We followed the PRISMA guidelines, helpful for the critical evaluation of scientific articles of various gynecological and extragynecological topics in order to keep clinicians up-to-date and provide the base for developing clinical trials or guidelines. Systematic reviews require collaboration of doctors with different specialties, strengthening the clinical and research team [287,553,554,555,556,557,558,559,560,561,562,563,564,565,566,567,568,569,570,571,572,573,574,575,576,577].

Limits of our study included the following: (1) the rarity and retrospective nature of available data (most metECs and PriCs were case reports), which did not allow for significant or reliable statistical analyses [6,7,8,9,10,11,12,13,14,15,16,17,18,19,20,21,22,23,24,25,26,27,28,29,30,31,32,33,56]; (2) incidence underestimation may be due to publication selection biases (authors’ choices of research topics; journal policies excluding case reports) [578]; (3) a lack of large multicenter studies or patients’ centralization, helpful for diagnostic and management purposes [579,580,581,582,583,584]; (4) the variable frequency of episiotomy procedure in different countries; (5) scant clinical–pathologic data (HPV testing, molecular analysis, etc.); (6) difficult assignment of the proper FIGO stage; (7) underestimation of other potential clinical–pathologic (co-)factors with prognostic impact. Future multicenter registries or collaborative studies could be helpful.

### 4.6. CC Management in Pregnancy

Cervical Pap smears should be preferably collected during the first prenatal visit; if positive, cervical biopsy can be performed. However, many women avoid gynecological exams. In some studies, pregnant and non-pregnant women have similar rates of abnormal Pap smears (2–8%); the cytological features can be altered by pregnancy-related changes, with potentially false-positive or negative results [80,81,187,188,189,231,269,585,586,587,588,589,590].

Pregnancy rates seem lower in oncologic patients, and placental metastases may occur, but the newborns in our study were healthy, without tumor metastases (when reported) [585,586,587,588,589,590,591,592,593,594,595,596,597,598,599,600,601,602]. Multidisciplinary management should deal with clinical and ethical dilemmas; in case of advanced-stage CCs, the decision to delay treatment until fetal maturity or to treat the patient immediately should consider gestational age, the patient’s will, CC size/stage, and life expectancy [585]. Before the 22nd gestational week, conization is recommended for HSIL (and maybe for early CCs); for a later diagnosis, surgery can be postponed to postpartum. Surgery is possible in all pregnancy trimesters but is best if performed during the early second trimester, with lower risk of miscarriage [185,192,194,195,205,239,254,258,259,586,587,588,589,590,603,604,605]. The NCCN guidelines [604] recommend a cesarean section ± radical hysterectomy and pelvic lymphadenectomy for pregnant stage I CC patients receiving delayed treatment. Radical trachelectomy successfully preserved pregnancy in few early-stage cases. Sentinel lymph node biopsy is contraindicated due to patent blue-induced anaphylactic shock or Tc-associated high radiation doses; if feasible, laparoscopic pelvic lymphadenectomy can be performed up to 22 gestational weeks due to technical issues. Traditional RT protocols should be modified, while ChT (usually contraindicated in pregnancy) was administered in exceptional cases [193,316,603,604,605,606,607,608,609,610,611,612,613,614].

Recent advances in immunotherapy (e.g., PD-1/PD-L1 inhibitors) may impact CC management. Indeed, increasing data from the literature and prospective studies demonstrated the effectiveness of immunotherapy or specific biomarker-specific treatments in CC or in tumors regardless of the histotype, thus promoting tumor-agnostic regulatory approaches [604]. For this reason, in recurrent, progressive, or metastastic patients, the NCCN guidelines recommend testing for mismatch repair system proteins (MMR), microsatellite instability, PD-L1 (positive for an immunohistochemical combined positive score, CPS ≥ 1), and comprehensive molecular profiling, including tumor mutation burden (TMB), *HER2*, *NTRK*, and *RET* analysis to select patients for immunotherapy and/or pan-tumor targeted drugs [272,604,615,616,617,618,619,620,621,622,623,624,625,626,627,628,629,630,631,632,633,634,635,636,637,638]. PD-1/PD-L1 targeted therapies achieved good results in immunologically “hot” tumors (non-small cell lung, renal, or bladder cancers, melanoma, etc.), while immunologically “cold” neoplasms can be resistant [639,640,641,642,643,644,645,646,647,648,649,650,651,652,653,654,655,656,657,658,659,660,661,662,663,664,665].

Pembrolizumab was approved for recurrent or metastatic PD-L1+ CCs (CPS ≥ 1) progressing on or after ChT, as well as for unresectable/metastatic, progressing solid tumors (highly instable, MMR deficient, or TMB-high) without other treatment options; pembrolizumab was included in NCCN-approved chemoradiation regimens for first- and second-line treatments of recurrent/metastatic disease and used in various mono- or combined-therapy options, sometimes in association with other drugs (such as platin-based drugs, topotecan, bevacizumab, tisotumab, etc.) and/or radiotherapy; other drugs affecting the PD-1/PD-L1 axis (cemiplimab, nivolumab) showed anti-CC activity and can be used in particular circumstances, but for further details, please refer to current NCCN guidelines [604,666,667,668,669,670,671,672]. Unfortunately, data about immunomarkers and immunotherapy were lacking in our series.

As metECs are rare, no clear guidelines are available for their management. An extensive gynecological examination is required, with accurate perineal and episiotomy inspection. Especially in pregnancy-related CC patients, careful and close follow-up and/or biopsy of new vulvar/perineal/episiotomy nodules or of pre-existing small lesions increasing in size are mandatory. When feasible, especially in otherwise low-stage tumors, metECs should be radically excised; during surgery, an attempt should be made to avoid anal injuries.

metECs were variably treated (surgery, ChT, and/or RT), with a good response in about half of the cases. Potential differences among various treatments in decreasing cancer recurrence should be further investigated. Large multicenter studies should be conducted to develop standardized protocols for reporting and managing these rare cases in the future.

## 5. Conclusions

1. As only 21 cases were reported, the episiotomy site seems to be a rare metastatic site; all metECs were pregnancy-associated CCs diagnosed in pregnancy or <1 year postpartum.
2. CC is the most common (72%) gynecologic cancer diagnosed in pregnancy, although its incidence is usually quite low (but variable as to the studied population).
3. Pregnancy may promote CC development and progression, but its prognostic impact should be further studied. CC can metastasize to episiotomy by spontaneous tumor detachment and implant, vaginal delivery, or iatrogenic manipulation (obstetrical or surgical), but vascular dissemination can also occur. These pathways may imply different metastatic risk; assignment of the proper stage could be difficult.
4. New episiotomy nodules (or pre-existing lesions increasing in size) should be carefully followed-up at short-term or biopsied; they may represent either benign lesions, PriCs, or metECs. Accurate clinical–radiological exams should identify and stage the primary tumor.
5. Especially in pregnancy-related CC patients, accurate gynecological exams should be conducted to search for episiotomy metECs.
6. Compared to PriCs, metECs occurred in younger (premenopausal) patients, were not associated with endometriosis, and demonstrated a slightly smaller size and a shorter mean time from episiotomy to episiotomy metastases, with a higher likelihood of a less favorable prognosis.
7. Cervical screening programs should be encouraged. Oncologic and obstetric CC treatment depends on gestational age, the patient’s choice, tumor stage and size, life expectancy, and the lymph node status. Before the 22nd gestational week, cervical conization for HSIL/early invasive CC is recommended; later, it may be appropriate to postpone surgery to postpartum. Surgery is best performed during the early second trimester, with lower risk of miscarriage. Cesarean section ± concurrent radical hysterectomy and pelvic node dissection are indicated for stage I CCs.
8. No treatment guidelines are available for metECs due to their rarity. The reported cases were variably treated (surgery and/or chemoradiation).

## Figures and Tables

**Table 1:** Carcinomas recurring in the episiotomy scar site: age, history, and diagnosis.

Case	Age	Parity	Pap Smear	Histological Diagnosis
1 [19]	38	G1P1	AGC, HPV16+ test (5 mo a.d.:)	ADC, Gr2 (usual type)
2 [20]	34	G1P1	ADC (2 mo a.d.)	ASC, Gr3 (glassy cell carcinoma)
3 [21]	35	G3P3A3	NR	ASC, Gr2
4 [22]	35	G2P1	autolyzed atypical epithelial cells (no malignant cells) (10 mo pre)	ASC, Gr3
5 [23]	36	G4P2A2	NR	SCC, Gr3
6 [24]	32	G3, P2002	AGC (7 mo pre); normal (4 mo pre)	ADC, Gr1 (villoglandular) (§, *)
7 [25]	35	multiparous	normal (early pregnancy, 35 weeks b.p.)	SCC, Gr2 (§)
8 [26]		NR	NR	NR (SCC or ADC)
9 [27]	26	G3P2	normal (early pregnancy and at 25 GW)	SCC, Gr1
10 [28]	29	G1	moderate dyskeratosis (17 GW) (previous year: normal)	ADC, Gr2-3 (§, *, °)
11 [29] (case 1)	37	NR	H-SIL (pre)	SCC, Gr3
12 [29] (case 2)	31	NR	NR	SCC, Gr3
13 [29] (case 3)	21	NR	NR	SCC, Gr3
14 [29] (case 4)	34	NR	NR	SCC, Gr3
15 [30]	32	G6P5A1	NR	SCC, Gr3
16 [17] (case 2)	29	G2P2	class IV (6 weeks a.d.)	SCC, Gr3
17 [17] (case 3)	30	G4P2	normal (pre and 9 weeks a.d.)	ADC, Gr3
18 [31]	24	P2002	normal (7 mo pre)	SCC
19 [32] (case 1)	32	G4P2A2	normal (pre)	ADC, Gr3
20 [32] (case 2)	32	G4P1A2	normal (pre)	ADC, Gr2 (*)
21 [33]	33	G1P1	occasional malignant cells (7 mo a.d.:)	SCC, Gr3 (°)

(§): microinvasive; (*): in situ adenocarcinoma was also evident; (°): H-SIL was also evident. a.d.: after delivery; ADC: adenocarcinoma; AGC: atypical glandular cell; ASC: adenosquamous carcinoma; Gr: grade; mo: months; GW: gestational week; H-SIL: high-grade squamous cell lesion; NR: not reported; pre: prenatal; SCC: squamous cell carcinoma.

**Table 2:** Carcinomas recurring in the episiotomy scar site: time sequence.

Case	CCD	ETD	CCD-ETD (mo)	CCD-PT (mo)	Delivery-PT (mo)	Last Delivery-ETD (mo)
1 [19]	PD (§)	SR	5	NR	NR	12
2 [20]	PD	P ($), DR	2	2	4	4
3 [21]	DD	SR	NR	NR	NR	5
4 [22]	DD (%)	DR	NR	0.75	0.75	1.25
5 [23]	PD (°)	P (°)	8	NR	NR	8
6 [24]	DD (*)	SR	NR	1	1	45
7 [25]	PD (*)	SR	0.25	NR	NR	66
8 [26]	NR	SR	NR	NR	NR	5
9 [27]	PD	P (&)	2 (&)	NR	2	1
10 [28]	PD (*)	SR	3	NR	3	4.25
11 [29]	DD	SR	NR	NR	0.5–2	2.25
12 [29]	DD (*)	SR	NR	NR	0.5–2	3
13 [29]	DD	SR	NR	NR	0.5–2	24
14 [29]	PD	SR	1.25	NR	0.5–2	2.25
15 [30]	PD	SR	2	NR	NR	5
16 [17]	PD	SR	1.5	4.5	6	7.5
17 [17]	PD (°)	P (°)	2.25	1	3.75	6.25
18 [31]	DD	SR	NR	11 days	11 days	1.25
19 [32]	PD	SR	3	NR	NR	NR
20 [32]	DD (*)	DR	NR	NR	NR	NR
21 [33]	PD	SR	7	3	10	30

(§): endocervical polyp found during last vaginal delivery (no histology); ($): 1st episiotomy lesion during surgery for cervical cancer; (%): tumor piece delivered with infant; (*): presentation during pregnancy [32], 3 months before [24], at 35th [25], or at 36th gestational week [29], while 1 case showed abnormal Pap smear and colposcopy during pregnancy [28]; (&): episiotomy tumor diagnosed before cervical cancer; (°): synchronous diagnosis of cervical cancer and episiotomy tumors. CCD: cervical cancer histological diagnosis; DD: diagnosis at delivery; DR: double recurrence; ETD: episiotomy tumor diagnosis; mo: months; NR: not reported; P: at presentation; PD: postpartum diagnosis; PT: primary treatment.

**Table 3:** Carcinomas recurring in the episiotomy scar site: clinical and follow-up data.

Case	CC Size (cm)	Episiotomy Tumor Size (cm)	FIGO Stage	Primary Treatment	Recurrence	Follow-Up (mo)
1 [19]	1.7	2.5	1b1	RH + BS + PELD(bi)	Episiotomy/perineum/vagina (right posterior), near external anal sphincter/puborectalis muscles (7 mo)	NED, 55
2 [20]	3	P: 5; R1: 5.5; R2: 9.8	3a/4 ($)	TAH + ENE + PELD(bi)	(1) Episiotomy/perineum (imaging re-evaluation after surgery); (2) large right inguinal LNs, vulva/perivulvar soft tissue (positive LN-FNAC, 2 mo later)	DOD, 9
3 [21]	NR	NR	1b	RH + RT	Episiotomy/perineum (5 mo)	NED, 120
4 [22]	5	R1: 6; R2: NR	1b3	Excision (CC) + TAH + PELD(bi) + ChT/RT	(1) Episiotomy and near OEU (18 days); (2) episiotomy and between OEU and clitoris, bilateral inguinal LNs, widespread intra-abdominal, sigma (obstruction, sigmoidostomy) (3.25 mo later)	DOD, 8
5 [23]	6	P: 4	3a/4 ($)	ChT(&)/RT	no	NED, 12
6 [24]	3 (*)	2	1a1	Polipectomy; TAH + PELD/PALD	(1) Episiotomy/perineum/left hemipelvis (positive FNAC) (44 mo); (2) left obturator LN (3 mo later)	NED, 48
7 [25]	4 (*)	5	1b (probable)	RH + PELD/PALD	Episiotomy (midline)/rectovaginal septum (66 mo)	NED, 120
8 [26]	NR	NR	2°	RT	Episiotomy (5 mo)	DOD, >5
9 [27]	NR	P: 4	3a/4 ($)	RT/BT, ChT (°, &)	Peri	DOD, 12
10 [28]	0.5 (*)	1.5	1a1	CB; TAH + PELD	(1) Episiotomy (6 weeks); (2) bilateral inguinal LNs, para-aortic LNs, liver, left lung base (2 mo later)	DOD, 16
11 [29]	NR	NR	1b/4b ($)	RH + PELD(bi)	Episiotomy (9 weeks)	DOD, 6
12 [29]	NR	NR	1b	RH + PELD(bi) (°)	Episiotomy (24 mo)	NED, 36
13 [29]	NR	NR	3c1	RH + PELD(bi)	Episiotomy (3 mo)	DOD, 36
14 [29]	NR	NR	3c1	RH + PELD(bi)	Episiotomy, pelvis (1 mo)	DOD, 6
15 [30]	NR	4	3b	RT + BT	Episiotomy (3 mo)	DOD, 7
16 [17]	7	P: 0.5	3a/4 ($)	VP + ENE + ChT (&) + RT + BT	No	AWD, NR
17 [17]	2	0.5	1b1	TAH + BSO + PELD(bi) (°)	Episiotomy (3 mo)	NED, 120
18 [31]	2	4	1b1	RH + PELD(bi)	Episiotomy/posterior fourchette (1 mo)	NED, 42
19 [32]	1	1	1b1	RH + BSO + PELD	Episiotomy	NED, 60
20 [32]	1	R1: 1; R2: 0.7	1b1	RH + PELD	(1) Episiotomy/lower rectovaginal septum (11 mo); (2) Episiotomy/perineum, pelvis, inguinal LNs (6 weeks later)	NED, 22
21 [33]	NR	4	1b	CB; RT/BT	Episiotomy/perirectal fat above the anorectal junction (20 mo)	AWD, 22

(*): polyp of 3 cm but tumor of 1.8 cm (invasion: 2 mm) [24]; minimal/2 mm stromal invasion [25,28]. (°): different first management (initial misdiagnosis: inflammation/benign): incision + antibiotics [27]; follow-up (9 months) [29]; podophyllin [17]. ($): unclear stage due to episiotomy [17,20,23,27] or maybe pulmonary [29] metastases at presentation. (&): notes for chemotherapy: cisplatinum, methotrexate, leucovirin, bleomycin (6 courses) (severe acute toxicity: stomatitis, alopecia, leukopenia) [27]; cisplatinum, methotrexate, bleomycin (3 courses) [17]; cisplatin [23]. Notes for RT/BT: cobalt, 50 Gy, whole pelvis, antero-posterior fields, midline block after 38 Gy [21]; RT (midplane 22 Gy in 10 fractions, 5 fractions/week, 2 anteroposterior–posteroanterior parallel opposing fields, 18 × 22 cm, perineum, bilateral inguinal areas) + BT (cesium, 3 uterovaginal insertions: 20 Gy in Manchester point A each; 1 cylindrical vaginal applicator: 30 Gy at 1 cm deep) [27]; external RT (5000 cGy) + BT (3500 mg-hr radium) [30]; 5 intracavitary courses, 15 cm^2^ field, 5 weeks [33]; BT (cesium) [17]. AWD: alive with disease; bi: bilateral; BS: bilateral salpingectomy; BSO: bilateral salpingo-oophorectomy; BT: brachytherapy; CB: cone biopsy; CC: cervical cancer; ChT: chemotherapy; DOD: dead of disease; ENE: episiotomy nodule excision; FNAC: fine-needle aspiration cytology; LN: lymph node; mo: months; NED: no evidence of disease; NR: not reported; OEU: orificium externum uerthrae; P: at presentation; PALD: para-aortic lymphadenectomy; PELD: pelvic lymphadenectomy; R1: 1st recurrence; R2: 2nd recurrence; RH: radical hysterectomy; RT: radiotherapy; TAH: total abdominal hysterectomy; VP: vaginal polypectomy.

**Table 4:** Treatment of recurrence, response to therapy, and side effects.

Case	Treament of Recurrence/PD (°)	Response to Therapy/Side Effects
1 [19]	RT, ChT (CP 40 mg/m^2^, weekly), BT	CR
2 [20]	(1) ChT (CP 65 mg/5 cycles), RT; (2) ChT (CP/TPC)	(1, 2) PD
3 [21]	Pelvic LN dissection (no tumor), RT, ChT (5-FU/MMC, 5 cycles), WE (no tumor)	CR. Post-ChT/RT side effects (<3 mo): dysparenunia, postcoital bleeding, rectoaginal fibrosis, proctitis, persistent perineal ulcer/necrosis, persistent tenesmus/rectal bleeding (*); after 10 years, recurrent perineal cellulitis and obstructive uropathy (bladder dysfunction, bilateral hydronephrosis, hydroureters, marked postvoid residual volumes)
4 [22]	(1) WE (20 days after hysterectomy; unclear surgical margins), RT, ChT (CP 50 mg/kg, 1.25 mo), BT; (2) palliative ChT, sigmoidostomy	PR
6 [24]	(1) RT (2) bi-SLN, bilateral groin LN dissection, partial radical vulvectomy (advancement of rhomboid flap) (free surgical margins)	CR
7 [25]	Excision (surgical margins: NR), RT, BT	CR. Post-RT vaginal stenosis
8 [26]	RT	NR
9 [27]	RT	PD. Good analgetic effect. Post-RT mucositis. Post-ChT stomatitis, leukopenia, alopecia
10 [28]	(1) WE (lymphovascular invasion close to free margins), BT; (2) ChT (bleomycin, ifosfamide, CP, 4 courses), RT	(1) CR; (2) PD. Post-ChT peripheral vasculitis, lethargy, dyspnea
11 [29]	ChT (MTX/VBL/A/CP)	NR
12 [29]	Follow-up (9 mo), ChT (MTX/VBL/A/CP), WE, RT	NR
13 [29]	RT, exenteration	NR
14 [29]	WE, RT	NR
15 [30]	ChT (CP/5-FU/A, 5 cycles), RT	PR
17 [17]	WE (unclear margins), RT, BT, WE (after 10 years, resection at the same site for suspected recurrence, but no residual tumor)	CR
18 [31]	WE (unclear margins), RT, BT (§)	CR. Post-RT chronic ulcer (§), rectal stricture
19 [32]	Excision (rectovaginal septum nodule), RT, BT	CR. Post-RT vaginal stenosis (vaginoplasty and split-thickness skin graft)
20 [32]	(1) WE (free margins); (2) excision (episiotomy nodules), RT	CR
21 [33]	Incomplete excision; abdomino-perineal (rectal)/posterolateral vaginal wall/episiotomy residual tumor resection (extraperitoneal end colostomy in left iliac fossa) (surgical margins status: unclear, probably free)	PR

Notes for RT/BT: Case 1: RT (external; pelvic primary lesion, cervical bed, obturator, internal/external iliac, presacral, common iliac, aortic bifurcation LNs; 50.4 Gy, 28 fractions) + BT (5 interstitial plastic catheters in vaginal wall, 21 Gy, 7 fractions, 2 fractions/day, 6 h interval; cobalt-60 high dose rate). Case 2: RT (whole pelvis, 24 Gy; perineum, 45 Gy). Case 3: RT (45 Gy, small anteroposterior photon fields inferior to the area demarcated by her prior treatment tattoos and fibrosis; 16.2 Gy then delivered using en face technique for perineal residual disease, total dose of 61.2 Gy). Case 4: RT (50/45 Gy, 25 doses). Case 6: RT (oppositional anterior–posterior portals, external iliac, lower internal iliac, and inguinal LNs; 1.8 Gy in 28 fractions, total dose of 50.4 Gy; 18 MV photons). Case 7: RT (45 Gy total dose; external beam RT (pelvis)) + BT (20 Gy high-dose-rate intracavitary vaginal boost, 4 doses of 5 Gy each). Case 9: palliative RT (14 Gy, 7 fractions, 2 oblique pelvic fields). Case 10: (1) BT (iridium wire implants), (2) palliative RT (inguinal area). Case 15: RT (2000 cGy external radiation to implantation site). Case 17: RT (external beam, perineal boost, Cobalt-60) + BT (radium, Delclos cylinders). Case 18: RT (whole pelvis, 4500 cGy, including perineal field in 25 fractions over 50 days) + BT (iridium192 implant, 1880 cGy to tumor volume, total minimum dose: 6380 cGy). Case 19: RT (5500 rad, 6 weeks; vulvar falloff whole-pelvic field, antero-posterior, and perineal port) + BT (vaginal cylinder radium, 2500 rad surface dose to upper vagina). Case 20: RT to pelvis, vulvar falloff, inguinal/pelvic LNs, 5500 rad. (°): Cases 5 and 16 did not recur, achieving CR [23] and PR [17], respectively. (*): treatment: sigmoid colectomy and diverting ileostomy. (§): wide resection of perineal ulcer and left gracilis musculocutaneous flap to reconstruct the perineal body (no residual tumor). 5-FU: 5-fluorouracil; A: adriamycin; bi-SLN: bilateral sentinel lymph nodes; BT: brachytherapy; ChT: chemotherapy; CP: cisplatin; CR: complete response; FNAC: fine-needle aspiration cytology; LN: lymph node; mo: months; MMC: mitomycin; MTX: metothrexate; NR: not reported; PD: progression of disease; PR: partial response; RT: radiotherapy; TPC: topotecan; VBL: vinblastine; WE: wide local excision.

**Table 5:** Carcinomas located at the episiotomy site: PriCs vs. metECs.

	PriCs (*n* = 13 Cases)	metECs (*n* = 21 Cases)
Histotype	2 (15%) SCC 1 (~8%) ACCBG 8 (62%) CCC 1 (~8%) EC 1 (~8%) SC (*)	21 (100%) SCC
Mean age (years)	50 (range: 31–70)	32 (range: 21–38)
Multiparity	6 (46%)	12 (75%)
Premenopausal	5 (39%)	21 (100%)
Peri-postmenopausal	5 (61%)	0 (0%)
Mean size (cm)	4.6 (range: 1–10)	3 (range: 0.5–6)
Endometriosis	8 (62%)	0 (0%)
HPV infection status	NR	1 (5%) HPV + 20 (95%) NR (probably HPV-related)
Mean time from episiotomy to first episiotomy tumor (months)	21 (range: 3–30)	12 (range: 12–792)
Lymph node metastases	3 (23%) pN+, 4 (31%) pN0	2 pN+, 6 pN0/pNx
Distant metastases	0 (0%)	Extra-episiotomy distant metastases were not clearly reported at presentation, maybe except for a case with unclear timing of lung metastases (presentation vs. recurrence)
Available follow-up data (months)	13 (100%) (range: 5–30; mean, 12)	21 (100%) (range, 6–120; mean, 40)
Recurrence rate	3 (23%)	19 (90%)
Time to recurrence	2 PD, 1 recurrence after 6 months	18 days to 66 months (mean, 12 months
Status at last follow-up (months)	11 (85%) NED (ERH: 5–15; mean, 7.8; EIC: 11–13; mean, 12) 2 (15%) DOD (1 CCC, 1 EC) (12–30)	9 (42%) DOD (6–36; mean, 12.5) 10 (48%) NED (12–120; mean, 63.5) 2 (10%) AWD

(*): diagnosed as a SC but probably representing a CCC or a EC. ACCBG: adenoid cystic carcinoma of Bartholin’s gland; AWD: alive with disease; CCC: clear cell carcinoma; DOD: dead of disease; EC: endometrioid carcinoma; EIC: endometriosis-independent carcinoma; ERH: endometriosis-related carcinoma; NED: no evidence of disease; NR: not reported; PD: progression of disease; PriCs: primary carcinomas of the episiotomy site; SC: serous carcinoma; SCC: squamous cell carcinoma.
